# A Review of Depth and Normal Fusion Algorithms

**DOI:** 10.3390/s18020431

**Published:** 2018-02-01

**Authors:** Doris Antensteiner, Svorad Štolc, Thomas Pock

**Affiliations:** 1 Center for Vision, Automation and Control, Austrian Institute of Technology, Vienna 1210, Austria; svorad.stolc@ait.ac.at (S.Š.); pock@icg.tugraz.at (T.P.); 2 Institute of Computer Graphics and Vision, Graz University of Technology, Graz 8010, Austria

**Keywords:** depth reconstruction, surface normals, optimization, Total Generalized Variation, primal-dual algorithm, computational imaging, least squares

## Abstract

Geometric surface information such as depth maps and surface normals can be acquired by various methods such as stereo light fields, shape from shading and photometric stereo techniques. We compare several algorithms which deal with the combination of depth with surface normal information in order to reconstruct a refined depth map. The reasons for performance differences are examined from the perspective of alternative formulations of surface normals for depth reconstruction. We review and analyze methods in a systematic way. Based on our findings, we introduce a new generalized fusion method, which is formulated as a least squares problem and outperforms previous methods in the depth error domain by introducing a novel normal weighting that performs closer to the geodesic distance measure. Furthermore, a novel method is introduced based on Total Generalized Variation (TGV) which further outperforms previous approaches in terms of the geodesic normal distance error and maintains comparable quality in the depth error domain.

## 1. Introduction

Measuring the depth of a scene accurately is essential for many tasks including applications in industrial environments, object recognition and security assurance. Usually the depth is measured by stereo cameras, structure from motion (SfM), time of flight (ToF) sensors, or light field cameras. These methods show accurate absolute depth maps but lack detail in high frequency depth structures. The reason for this lies in the dependency on the presence of structural information in the image as well as in the analysis routine, which is usually done by hypothesis testing and therefore limited in range and step sizes. Stereo matching methods include correlation based techniques [1], semi global matching [2,3], block based matching [4,5] and stereo matching for micro array cameras [6]. Several methods were introduced to retrieve depth information from light field data using epipolar image slope analysis [7], structure tensors [8], fine-to-coarse approaches [9,10] and by line consistency metrics [11]. Structure from motion and time of flight techniques were presented in [12,13,14], respectively. In contrast to depth based approaches, methods that recover surface normals, such as photometric stereo [15], show high frequency details but lack an absolute depth reference. Shape from shading was used to retrieve surface normals by [16]. A robust normal reconstruction using photometric stereo information with a Markov Random Field (MRF) was introduced in [17]. Previous methods have been presented, which retrieve surface normals from a calibrated stereo setup. This can be achieved e.g., by estimating the homography between two matched patches [18,19] or by using the affine transform data between two projections [20] additionally to the reconstruction of a sparse depth map retrieved from a stereo correspondence analysis. A learning-based method using a tandem of convolutional neural networks to estimate depth and surface normals from image data simultaneously was introduced in [21]. Combining depth and surface normal data allows precise depth reconstructions for low- as well as high-frequency components in the depth map.

Depth maps and surface normals were previously combined in various ways. Shape from shading was used under general illumination in [22], photometric stereo normals were incorporated in [23,24,25]. Another approach was presented in [26], where the tangent plane of the given normals was projected into the measured normal field. This normal constraint was previously used in several algorithms (e.g., [27,28,29,30]). The method described in [31] uses a standard depth constraint and forces the Laplacian of the optimal solution to be in the proximity of the derivative of the given normals. In [32] a depth and photometric stereo fusion algorithm was introduced, which uses additional Laplacian smoothing term and adaptive pixel-wise weighting parameters to preserve surface discontinuities. The Laplacian smoothing term was also added in [33]. A extended penalty is chosen in [34], where the normal is enforced to be close to the normal from the initial depth map, while 2nd-order spherical harmonics are used to constrain the normals according to the observed shading in the input image, a smoothness function enforces the similarity of 1st-order neighbors and an additional term constrains the normals to unit length. A method to refine depth by photometric stereo information using RGB-D cameras was introduced in [35], where an energy function is optimizing for the depth, smoothness, shading and temporal aliasing of a scene. Surface normals from polarization cues were used to enhance the depth map in [36], in an iterative process the depth is refined with corrected surface normal information and a depth fidelity constraint, which enforces consistency between the surface from normals and accurate regions in the depth map. An original approach for inferring about the surface normals from light field data as well as a hybrid setup combining depth maps with surface normals using a block coordinate descent algorithm was demonstrated in [37].

Even though several methods to combine surface depth with surface orientation data previously emerged, a thorough analysis and classification of the properties of those approaches was missing. In this paper, our first main contribution comprises an in-depth comparison of several variational methods using depth maps and surface orientation data, as well as a classification and evaluation of weighting terms for surface orientations using gradients or surface normals. We analyze orientation weighting terms of common methods and explain their differences in respect to the geodesic distance weighting. We show that methods which behave closer to this natural surface normal weighting term show a better performance, especially in regions with steep depth edges. Based on the findings we introduce our second main contribution, a new generalized formulation of a previously introduced method [26], which outperforms other methods regarding the error in the depth domain. Our third main contribution is a novel gradient-based approach, which is using TGV and outperforms other methods in the domain of the geodesic error of the resulting normals.

## 2. Depth and Surface Normal Cues

At present, 3D models are used for a wide range of analysis tasks. Depth models are being constructed by acquisition devices using stereo systems, light field cameras, time of flight (ToF), or other range scanning techniques. Common methods show a high precision in the absolute depth measurement, but a low quality in fine relative details. These errors are major obstructions for tasks such as finding defects in objects. Measuring the normal fields of objects by using methods such as photometric stereo [15] or shape from shading [38] will allow the reconstruction of surfaces with highly precise local details. On the downside, those methods show errors in the low frequency domain and therefore result in a low absolute depth accuracy.

Combining depth maps with surface normal information allows an exact 3D reconstruction both in absolute depth and fine surface details. This can be achieved by optimizing energy functions by variational methods, where the solution is penalized for deviating from the depth model and from the surface normals. In state-of-the-art techniques, the surface normal component is either used directly or by converting it to gradient information, where the *x*- and *y*-component can be treated independently. Such an independent treatment can be beneficial for applications where data components are missing, as for example line-scanners [39].

## 3. Notations and Preliminaries

In this section, we introduce the essential notations used across this paper. By default we assume discretized surface structures of the size of M×N pixels. In order to access the image location, we define the following index set I={(i,j):1≤i≤M,1≤j≤N}.

The discrete depth map of our scene is scalar valued in each pixel and defined as follows:

(1) $$Z={\left(Z_{i,j}\right)}_{i,j\in I}\in R^{M\times N}.$$

Variables with a bold font refer to surface structures where each pixel is vector valued. Hence, the surface gradient field G in *x*- and *y*-direction is defined as:

(2) $$G={\left(G_{i,j}\right)}_{i,j\in I}\in R^{M\times N\times 2},\;\mathrm{where}\;G_{i,j}=\left(G_{i,j,x},G_{i,j,y}\right).$$

The gradient of the depth map is computed using standard finite differences:

(3) $$\nabla Z={\left({\left(\nabla Z\right)}_{i,j}\right)}_{i,j\in I},\;\mathrm{where}\;{\left(\nabla Z\right)}_{i,j}=\left({\left(\nabla_{x}Z\right)}_{i,j},{\left(\nabla_{y}Z\right)}_{i,j}\right),$$

and the gradient operator in *x*- and *y*-direction $\nabla :R^{M\times N}\to R^{M\times N\times 2}$ is given by:

(4) $$\begin{array}{c} {\left(\nabla_{x}Z\right)}_{i,j}=\left\{\begin{array}{cc} Z_{i+1,j}-Z_{i,j} & \;\mathrm{if}\;1\leq i<M,\\ 0, & \mathrm{otherwise}, \end{array}\right.\\ {\left(\nabla_{y}Z\right)}_{i,j}=\left\{\begin{array}{cc} Z_{i,j+1}-Z_{i,j} & \;\mathrm{if}\;1\leq j<N,\\ 0, & \mathrm{otherwise}. \end{array}\right. \end{array}$$

The surface normal field is defined as follows:

(5) $$N={\left(N_{i,j}\right)}_{i,j\in I}\in R^{M\times N\times 3},\;\mathrm{where}\;N_{i,j}=\left(N_{i,j,x},N_{i,j,x},N_{i,j,z}\right)\in R^{3}.$$

By definition, we have $|N_{i,j}{|}_{2}=1$. The relation between the surface gradient estimation and the surface normals is defined for all i,j∈I as follows:

(6) $$N_{i,j,x}=\frac{-{\left(\nabla_{x}Z\right)}_{i,j}}{|\left(-{\left(\nabla Z\right)}_{i,j},1\right){|}_{2}},\;N_{i,j,y}=\frac{-{\left(\nabla_{y}Z\right)}_{i,j}}{|\left(-{\left(\nabla Z\right)}_{i,j},1\right){|}_{2}},\;\mathrm{and}\;N_{i,j,z}=\frac{1}{|\left(-{\left(\nabla Z\right)}_{i,j},1\right){|}_{2}}.$$

Furthermore, we are using two specific surface tangent vectors, which are aligned with the *x*- and *y*-vector respectively and defined as follows:

(7) $$T={\left(T_{i,j}\right)}_{i,j\in I}\in R^{M\times N\times 2\times 3},\;\mathrm{where}$$

(8) $$T_{i,j}=\left(T_{i,j,x},T_{i,j,y}\right)\;\mathrm{and}$$

(9) $$T_{i,j,x}=\left(1,0,{\left(\nabla_{x}Z\right)}_{i,j}\right)\;\mathrm{and}\;T_{i,j,y}=\left(0,1,{\left(\nabla_{y}Z\right)}_{i,j}\right).$$

## 4. Depth and Surface Normal Fusion Algorithms

**Figure 1:**
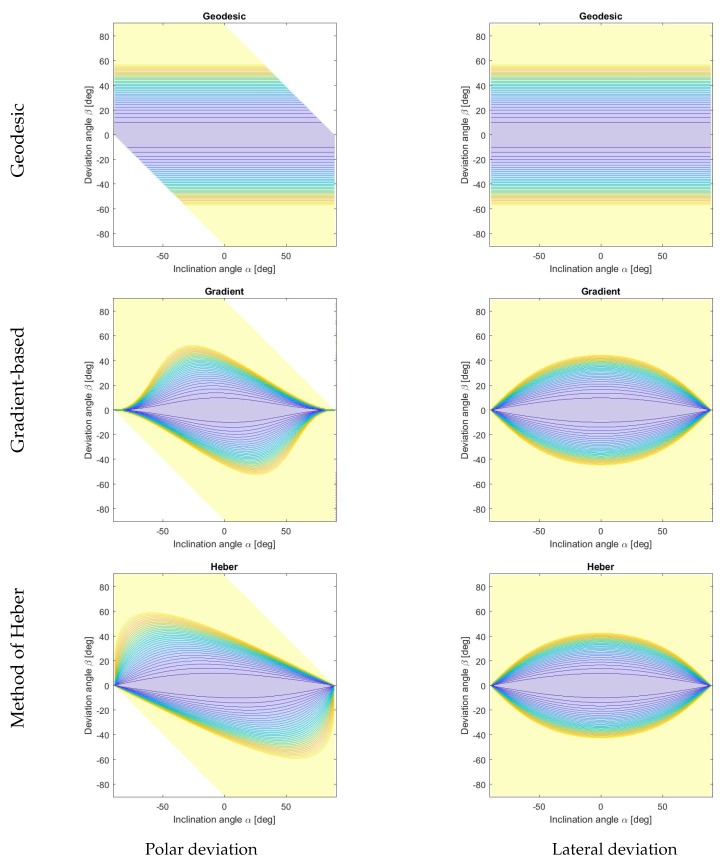
Quadratic penalty functions of the surface orientation constraint visualized for deviations in polar (left column) and lateral (right column) directions. Shown are the geodesic energy (top row), the gradient-based method (middle row) and the method of Heber (bottom row). Colors of the contours indicate the respective penalty values obtained for different combinations of the inclination and deviation angles. Note that the range is clipped between 0 (blue) and 1 (yellow). See Figure 3 for the explanation of the parametrization used.

**Figure 2:**
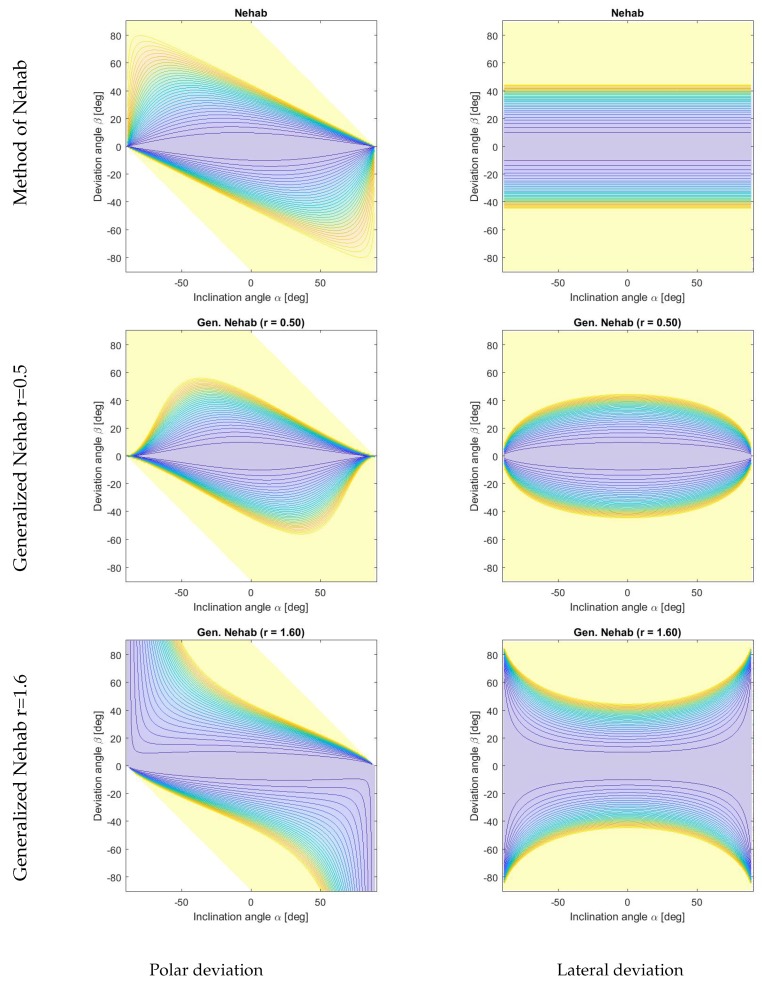
Quadratic penalty functions of the surface orientation constraint visualized for deviations in polar (left column) and lateral (right column) directions. Shown are the method of Nehab (top row), the generalized method of Nehab with *r* = 0.5 (middle row) as well as *r* = 1.6 (bottom row). A more natural weighting, which is in the proximity of the geodesic distance, can be achieved by the adaption of the parameter *r*. A well balanced distance measure such as with r=1.6 has the potential of performing better in steep regions (high inclination angle) while preserving performance in the flat regions. Colors of the contours indicate the respective penalty values obtained for different combinations of the inclination and deviation angles. Note that the range is clipped between 0 (blue) and 1 (yellow). See Figure 3 for the explanation of the parametrization used.

**Figure 3:**
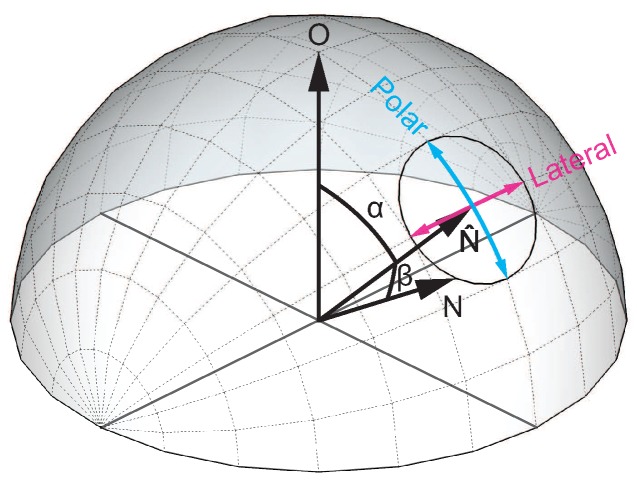
The distance between a two surface normals $\hat{N}$ and N is expressed by means of the horizontal polar coordinate system. The distances in Figure 1 and Figure 2 are measured in lateral and polar directions using angles α and β. The former describes the angle between the given normal $\hat{N}$ and the the upright vector O, the latter defines the angular deviation between the normals $\hat{N}$ and N.

In this paper, we analyze state-of-the-art methods in a systematic way and introduce two novel approaches. The described hybrid depth and surface normal methods are categorized in terms of their penalty functions. State-of-the-art approaches used similar depth penalty terms and differed in the surface orientation weighting and regularization. We organize the described methods in two categories: (i) gradient-based and (ii) normal-based approaches as well as with respect to their treatment of flat and steep surface regions. While the presented methods show similar behaviors in flat areas, they differ in the penalization of steep regions. We show the quadratic penalty functions of the methods presented in Figure 1 and Figure 2 for lateral and polar deviations, which are illustrated in Figure 3. We will show that the behavior of the energy function for different inclination angles correlates with the quantitative and qualitative depth reconstruction performance of each individual method. We argue that the geodesic distance function shows the most natural behavior with the favorable property of penalizing the distance of the normal orientation independent of the steepness of the edges. Due to this ideal behavior, we use the function to evaluate other distance measures in Section 5. Without loss of generality, we assume a dense depth and normal map for the algorithms described in this paper. In case of sparse input data, we suggest an extension based on Poisson surface reconstruction [40] to deal with sparse data.

An overview of the presented methods is given in Table 1. The first method we present in Section 4.2 is the construction of depth from only the gradient surface orientation information (i.e., no prior information about the absolute depth is being used). Using only the surface orientation for the depth reconstruction results in large-scale low-frequency errors (and therefore depth offsets). Later we overcome this problem by introducing an additional depth constraint in all following methods.

Second, we introduce the gradient-based approach with a depth constraint formulated as a least squares problem in Section 4.3. The respective contour plot of the orientation distance measure shows a strong penalization of steep edges in contrast to flat surface regions. It is easy to see (Figure 1), that the error from the same angular deviation due to noisy normals may generate from small up to infinity error in the gradient domain, depending on the inclination angle. For demonstrative purposes, we additionally show two extensions of the gradient-based method with regularization terms. One forces gradient-based smoothing. The other one enforces smoothness with a Laplacian term and can be used for the reconstruction with sparse depth and surface normal data.

Third, the method of Heber [41] for combining depth with surface orientations is shown in Section 4.4, which scales the given normal by the length of the optimized gradient.

Forth, a review of the method of Nehab [26] is given in Section 4.5, which reprojects the tangents of the optimized surface onto the given normal.

Fifth, as one of our main contributions in this paper, we introduce a new penalty function in form of a generalized method of Nehab in Section 4.6. Using a novel parametrization moves the penalty function closer to the geodesic normal energy and hence penalizes deviations in steep edges and flat regions more equally.

Last, we introduce our second main contribution, a novel Total Generalized Variation (TGV) model in Section 4.7, which penalizes the distance of the gradients of the surface orientation and gives significantly improved reconstruction results. The reason for this lies in the decoupling of the gradient through the TGV term.

### 4.1. Geodesic Distance

In the 3D space, the geodesic distance is the most natural surface normal penalty as distances between surface normals are weighted equally, independent of the surface slope angle with respect to the observer. Therefore it is used in this paper as a comparison measure for the evaluation in Section 5.

The geodesic distance $d_{i,j}$ is defined as the inverse cosine of the point-wise dot product between the given normal $\hat{N}\in R^{M\times N\times 3}$ and the estimated solution normal field $N\in R^{M\times N\times 3}$ as follows:

(10) $$d_{i,j}=acos\left(\langle {\hat{N}}_{i,j},N_{i,j}\rangle \right),\;\forall i,j\in I,$$

where $\langle \cdot ,\cdot \rangle$ denotes the standard dot product, which is defined as $\langle a,b\rangle =\sum_{i=1}^{n}a_{i}\;b_{i}=||a||\;||b||\;cos\left(\phi \right)$ and ϕ describes the angle between *a* and *b*. We can formulate the distance in Equation (10) by utilizing the surface gradient estimation $\nabla Z\in R^{M\times N\times 2}$ for the surface normal N with the relations shown in Equation (6) as follows:

(11) $$d_{i,j}=acos\left(\frac{\langle {\hat{N}}_{i,j},\left(-{\left(\nabla Z\right)}_{i,j},1\right)\rangle }{|\left(-{\left(\nabla Z\right)}_{i,j},1\right){|}_{2}}\right).$$

The surface orientation weighting of the geodesic distance is illustrated in the contour plot in the first row of Figure 1. The first column shows the polar deviation of the coordinates and the second column shows the lateral deviation, parameterized by the inclination angle α and the deviation angle β, as illustrated in Figure 3. For the following methods a balanced weighting over all inclination angles both for the polar and lateral deviation is favored, as provided by the geodesic distance.

### 4.2. Gradient-Based Method with Surface Orientation Constraint Only

As typical for photometric stereo methods, depth can be partly recovered from surface orientations only. In order to provide a complete context for the method considered in this paper, we show here a method that is using solely gradient-based data. Gradient-based methods have previously been frequently utilized for depth reconstruction (e.g., [16,42,43,44,45,46,47]). Given a gradient field $\hat{G}\in R^{M\times N\times 2}$, we calculate the surface gradients for the estimated depth map *Z* in *x*- ($\nabla_{x}Z$) and *y*-direction ($\nabla_{y}Z$) respectively. Combining relations from Equation (6), the relations of surface normals and gradients are as follows:

(12) $$|\left(-{\left(\nabla Z\right)}_{i,j},1\right){|}_{2}=-\frac{{\left(\nabla_{x}Z\right)}_{i,j}}{N_{i,j,x}}=-\frac{{\left(\nabla_{y}Z\right)}_{i,j}}{N_{i,j,y}}=\frac{1}{N_{i,j,z}},\;\forall i,j\in I,$$

hence the surface gradients are given as:

(13) $${\left(\nabla_{x}Z\right)}_{i,j}=-\frac{N_{i,j,x}}{N_{i,j,z}}\;\mathrm{and}\;{\left(\nabla_{y}Z\right)}_{i,j}=-\frac{N_{i,j,y}}{N_{i,j,z}},\;\forall i,j\in I,$$

and the given gradient fields correspond to the surface normals by:

(14) $${\hat{G}}_{i,j,x}=-\frac{{\hat{N}}_{i,j,x}}{{\hat{N}}_{i,j,z}}\;\mathrm{and}\;{\hat{G}}_{i,j,y}=-\frac{{\hat{N}}_{i,j,y}}{{\hat{N}}_{i,j,z}},\;\forall i,j\in I,$$

in *x*- and *y*- direction respectively. Our goal is to compute a depth map *Z* such that

(15) $${\left(\nabla Z\right)}_{i,j}\approx {\hat{G}}_{i,j},\;\forall i,j\in I.$$

**Figure 4:**
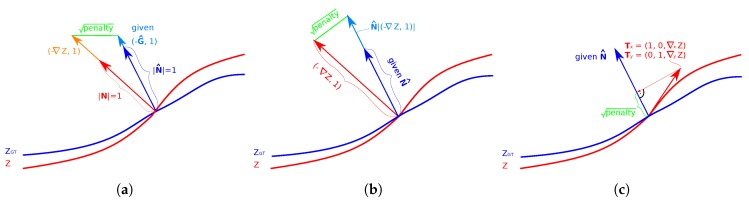
Comparison of the penalty of (**a**) the gradient-based method; (**b**) the method of Heber and (**c**) the method of Nehab. Gradient-based method: comparison of the measured gradient field components $\left(-\nabla_{x}Z,-\nabla_{y}Z\right)$ with the given gradient field $\left(-{\hat{G}}_{x},-{\hat{G}}_{y}\right)$. Method of Heber: scaling of the normal vector $\hat{N}$ by the length of $\left(-\nabla_{x}Z,-\nabla_{y}Z,1\right)$. Method of Nehab: distance by projection of the tangents $T_{x}$ and $T_{y}$ of the optimized surface onto the given normal field $\hat{N}$.

The comparison of the resulting penalty between the measured and the given gradients is illustrated in Figure 4a. Since Equation (15) is an overdetermined system of linear equations, it can be solved as the following least squares problem:

(16) $$\min_{Z}\frac{1}{2}||\nabla Z-\hat{G}{\left|\right|}^{2},$$

whose global minimizer $Z_{min}$ satisfies the first order sufficient optimality condition:

(17) $$\nabla^{*}\left(\nabla Z_{min}-\hat{G}\right)=0,$$

where $\nabla^{*}:R^{M\times N\times 2}\to R^{M\times N}$ denotes the adjoint of the ∇ operator, with $\nabla^{*}=\left(\nabla_{x}^{*},\nabla_{y}^{*}\right)$. We compute the minimizer using a standard conjugate gradient method.

It is well known that reconstructing the depth using only surface normal data usually results in errors in the low frequency domain. In the past, this has been improved by different approaches, such as introducing additional boundary conditions [44]. We resolve this problem by hybrid depth and surface normal formulations which proved very efficient in finding an accurate surface reconstruction.

### 4.3. Gradient-Based Method

In this section, we discuss a hybrid gradient-based method which reconstructs depth using a gradient-based algorithm (similar to the one from Section 4.2) extended by the use of the given initial depth $\hat{Z}$. Also here the gradient of the estimated depth map ∇Z is forced to be in the proximity of a measured gradient $\hat{G}$ in *x*- and in *y*-direction. Therefore, we formulate an overdetermined system of equations as follows:

(18) $$Z\approx \hat{Z}\;\mathrm{and}\;{\left(\nabla Z\right)}_{i,j}\approx {\hat{G}}_{i,j},\;\forall i,j\in I.$$

The corresponding least squares problem is given as:

(19) $$\min_{Z}\frac{1}{2}||Z-\hat{Z}{\left|\right|}^{2}+\frac{\lambda }{2}||\nabla Z-\hat{G}{\left|\right|}^{2},$$

where λ>0 is used to balance between the depth and the orientation constraints. The global optimizer $Z_{min}$ is found by a standard conjugate gradient method, with the optimality condition given as follows:

(20) $$Z_{min}-\hat{Z}+\lambda \nabla^{*}\left(\nabla Z_{min}-\hat{G}\right)=0.$$

The contour plots of the surface orientation penalty function corresponding to the gradient-based method are shown in Figure 1. Note that with this method, the penalty is notably stronger for deviations in steep regions than in flat regions.

For demonstrative purposes, we introduce the extension of the gradient-based method in Equation (19) with a regularization term. We use a Laplace 2nd-order method which is driven by the derivative of the given gradient field. We formulate the following least squares problem:

(21) $$\min_{Z}\frac{1}{2}||Z-\hat{Z}{\left|\right|}^{2}+\frac{\lambda }{2}||\nabla Z-\hat{G}{\left|\right|}^{2}+\frac{\lambda_{R}}{2}||\Delta Z-\left(-\nabla^{*}\hat{G}\right){\left|\right|}^{2},$$

where $\lambda_{R}>0$ balances the regularization, $\nabla^{*}\hat{G}$ can be decomposed into $\left(\nabla_{x}^{*}\hat{G_{x}},\nabla_{y}^{*}\hat{G_{y}}\right)$ and Δ denotes the Laplace operator which is defined as follows:

(22) $$\Delta_{x}=-\nabla_{x}^{*}\nabla_{x}\;\mathrm{and}\;\Delta_{y}=-\nabla_{y}^{*}\nabla_{y}.$$

Such a gradient-based regularization can be applied to all presented methods.

The presented gradient-based approaches require dense depth and surface orientation data. A possibility to cope with sparse data is an additional smoothness assumption coupled with pixel-wise weighting parameters. Hence, we add another term to Equation (19) with a Laplacian smoothness assumption as follows:

(23) $$\min_{Z}\frac{1}{2}\left|\right|\lambda_{1}⊙\left(Z-\hat{Z}\right){\left|\right|}^{2}+\frac{1}{2}\left|\right|\lambda_{2}⊙\left(\nabla Z-\hat{G}\right){\left|\right|}^{2}+\frac{1}{2}\left|\right|\lambda_{3}⊙\left(\Delta Z\right){\left|\right|}^{2}.$$

The weighting parameters $\lambda^{1}$ and $\lambda^{2}$ can be given a priori by the confidence of a data point and $\lambda^{3}$ by the inverse confidence and are defined as follows:

(24) $$\begin{array}{ccc} \lambda_{1} & = & {\left(\lambda_{1,i,j}\right)}_{i,j\in I}\in R^{M\times N}, \end{array}$$

(25) $$\begin{array}{ccc} \lambda_{2} & = & {\left(\lambda_{2,i,j}\right)}_{i,j\in I}\in R^{M\times N\times 2},\;\mathrm{where}\;\lambda_{2,i,j}=\left(\lambda_{2,i,j,x},\lambda_{2,i,j,y}\right),\;\mathrm{and} \end{array}$$

(26) $$\begin{array}{ccc} \lambda_{3} & = & {\left(\lambda_{3,i,j}\right)}_{i,j\in I}\in R^{M\times N\times 2},\;\mathrm{where}\;\lambda_{3,i,j}=\left(\lambda_{3,i,j,x},\lambda_{3,i,j,y}\right). \end{array}$$

In case of stereo or light-field methods for depth reconstruction the parameters can be assessed base on the matching confidence. Unknown points would have a confidence of zero. The same extension for sparse data is applicable for all following methods. Therefore, without loss of generality we discuss the weighting of the surface orientation term with a focus on dense depth and surface orientation data without an additional smoothness assumption.

### 4.4. The Method of Heber

A hybrid variational refinement model was described by Heber [41], where an initial rough depth $\hat{Z}$ is given and refined with surface normal information:

(27) $$\min_{Z}\frac{1}{2}||Z-\hat{Z}{\left|\right|}^{2}+\frac{\lambda }{2}||\left(-\nabla Z,1\right)-\hat{N}⊙{\left|\left(-\nabla Z,1\right)\right|}_{2}|{|}_{2}^{2},\;\mathrm{where}$$

(28) $${\left|\left(-\nabla Z,1\right)\right|}_{2}=\left(|\left(-{\left(\nabla Z\right)}_{1,1},1\right){|}_{2},|\left(-{\left(\nabla Z\right)}_{1,2},1\right){|}_{2},\ldots ,{\left|\left(-{\left(\nabla Z\right)}_{M,N},1\right)\right|}_{2}\right)$$

defines the vector of pointwise 2-norms and the symbol ⊙ denotes the operator for the point-wise product, also known as Hadamard product. This method is conceptually similar to the gradient-based method described in Section 4.3, also here the same depth constraint ensures a result in the proximity of an initial depth solution $\hat{Z}$. However, the surface orientation constraint used in Heber’s method exploits the given normal field $\hat{N}$ directly instead of the gradient field $\hat{G}$. Here the normalization of ∇Z by division by the length of the vector is overcome by multiplying the term on one side by ${\left|\left(-\nabla Z,1\right)\right|}_{2}$, which leads to a convex problem [41].

An illustration of the comparison of the measured ∇Z and the given normal $\hat{N}$ is shown in Figure 4b. The given normal is scaled to the length of the measured gradients ∇Z. The contour map in Figure 1 demonstrates the normal weighting of the Heber’s energy term. Here, similar to the gradient-based method, a deviation in steep edges is penalized more than a deviation in flat regions. Also, a different weighting applies whether the estimated gradients are steeper or flatter than the given values.

As the energy function from Equation (27) is convex and differentiable, this algorithm can be solved by an (accelerated) gradient descent method or a (fast) proximal gradient approach. For our evaluation, we used a plain gradient descent approach.Nesterov [48] proposed an accelerated gradient descent method with a simple weighted gradient step, followed by additional sliding, based on the last estimation. A fast proximal gradient method has an additional extrapolation step compared to the proximal gradient method. An example is the Fast Iterative Shrinkage Thresholding Algorithm (FISTA) [49] (see Appendix A.1).

### 4.5. The Method of Nehab

The method of Nehab [26] combines depth and surface normals by solving a system of linear equations, consisting of depth and surface orientation constraints. This method is similar to the gradient-based method described in Section 4.3, only the surface normal information is leveraged in a different way, making different trade-offs between flat and steep gradients.

In this method, the surface normal constraint optimizes the sum of squared projections of a set of surface tangent vectors T, as defined in Equation (7) through Equation (9), of the reconstructed surface *Z* onto the given normal field $\hat{N}$. This surface normal penalty has been adopted previously in several approaches (e.g., [21,27,28,29,30]). The projection is illustrated in Figure 4. Note that the lowest penalty 0 is reached when the tangent vector is precisely orthogonal to the given normal vector $\hat{N}$. We first consider the formulation as described by Nehab [26] by formulating an overdetermined linear system of sparse equations:

(29) $$\begin{array}{ccc} Z & \approx & \hat{Z},\\ \langle {\hat{N}}_{i,j},T_{i,j,x}\rangle ={\hat{N}}_{i,j,x}+{\hat{N}}_{i,j,z}{\left(\nabla_{x}Z\right)}_{i,j} & \approx & 0,\;\mathrm{and}\\ \langle {\hat{N}}_{i,j},T_{i,j,y}\rangle ={\hat{N}}_{i,j,y}+{\hat{N}}_{i,j,z}{\left(\nabla_{y}Z\right)}_{i,j} & \approx & 0,\;\forall i,j\in I, \end{array}$$

which leads to the following least squares problem:

(30) $$\begin{array}{c} \min_{Z}\frac{1}{2}||Z-\hat{Z}{\left|\right|}^{2}+\frac{\lambda }{2}||{\hat{N}}_{z}⊙\nabla_{x}Z+{\hat{N}}_{x}|{|}^{2}+\frac{\lambda }{2}||{\hat{N}}_{z}⊙\nabla_{y}Z+{\hat{N}}_{y}|{|}^{2} \end{array},$$

where the parameter λ∈[0,1] weights the influence of the initial depth and the given normals. The global optimizer $Z_{min}$ is calculated with a standard conjugate gradient method, with the optimality condition given as follows:

(31) $$Z_{min}-\hat{Z}+\lambda \left(\nabla_{x}^{*}\left({\hat{N}}_{z}⊙\left({\hat{N}}_{z}⊙\nabla_{x}Z_{min}+{\hat{N}}_{x}\right)\right)+\nabla_{y}^{*}\left({\hat{N}}_{z}⊙\left({\hat{N}}_{z}⊙\nabla_{y}Z_{min}+{\hat{N}}_{y}\right)\right)\right)=0.$$

The weighting of the surface normal information is explained in more detail in Figure 4c and further illustrated in a contour plot in Figure 2. The polar deviation to the given surface orientation in steep and flat regions is penalized similarly to the method of Heber (see Figure 1, bottom row, left). However, the lateral deviations are weighted equally which is the same behavior as the ideal geodesic distance function (see Figure 1, top row, right).

Note that the approach of Nehab, as described in Equation (29), corresponds to the gradient-based method with an additional local $N_{z}$-weighting applied to both sides of the surface normal constraint of Equation (18). This can be shown by utilizing the equivalences defined in Equations (13) and (14) as follows:

(32) $${\hat{N}}_{i,j,x}={\hat{N}}_{i,j,z}\;{\left(\nabla_{x}Z\right)}_{i,j}\;\mathrm{and}\;{\hat{N}}_{i,j,y}={\hat{N}}_{i,j,z}\;{\left(\nabla_{y}Z\right)}_{i,j},\;\forall i,j\in I,$$

and formulating a corresponding overdetermined linear system of equations:

(33) $$\begin{array}{ccc} Z & \approx & \hat{Z},\\ {\hat{N}}_{i,j,z}\;{\left(\nabla_{x}Z\right)}_{i,j} & \approx & {\hat{N}}_{i,j,z}\;{\hat{G}}_{i,j,x},\;\mathrm{and}\\ {\hat{N}}_{i,j,z}\;{\left(\nabla_{y}Z\right)}_{i,j} & \approx & {\hat{N}}_{i,j,z}\;{\hat{G}}_{i,j,y},\;\forall i,j\in I. \end{array}$$

Compared with the gradient-based method, the $N_{z}$-weighting inherent to Nehab’s method improves the behavior of the penalty function by weakening the influence of regions with steep edges. Rationale behind the $N_{z}$-weighting follows from the fact that flat regions exhibit a higher value of $N_{z}$ (close to 1) while steep regions receive low $N_{z}$ values (close to 0). Such a local weighting helps preventing the over-penalization in steep regions as observed in the gradient-based method.

### 4.6. Generalized Nehab

In order to allow the normal weighting to reach a closer proximity to the geodesic distance, we propose a generalization of the method of Nehab. We extend on the concept of the gradient $N_{z}$-weighting as explained in Equation (33) by introducing an additional exponent *r* that controls influence of the $N_{z}$-weighting such that:

(34) $$\begin{array}{ccc} Z & \approx & \hat{Z},\\ {\left({\hat{N}}_{i,j,z}\right)}^{r}\;{\left(\nabla_{x}Z\right)}_{i,j} & \approx & {\left({\hat{N}}_{i,j,z}\right)}^{r}\;{\hat{G}}_{i,j,x},\;\mathrm{and}\\ {\left({\hat{N}}_{i,j,z}\right)}^{r}\;{\left(\nabla_{y}Z\right)}_{i,j} & \approx & {\left({\hat{N}}_{i,j,z}\right)}^{r}\;{\hat{G}}_{i,j,y},\;\forall i,j\in I. \end{array}$$

We optimize the corresponding least squares problem with a standard conjugate gradient method:

(35) $$\min_{Z}\frac{1}{2}||Z-\hat{Z}{\left|\right|}^{2}+\frac{\lambda }{2}||{\left({\hat{N}}_{z}\right)}^{r}⊙\nabla_{x}Z-{\left({\hat{N}}_{z}\right)}^{r}⊙{\hat{G}}_{x}|{|}^{2}+\frac{\lambda }{2}||{\left({\hat{N}}_{z}\right)}^{r}⊙\nabla_{y}Z-{\left({\hat{N}}_{z}\right)}^{r}⊙{\hat{G}}_{y}|{|}^{2},$$

where we find a global optimizer $Z_{min}$ that satisfies the following optimality condition:

(36) $$\begin{array}{cc} Z_{min}-\hat{Z}+\lambda (\nabla_{x}^{*}\left({\left({\hat{N}}_{z}\right)}^{r}⊙\left({\left({\hat{N}}_{z}\right)}^{r}⊙\nabla_{x}Z_{min}-{\left({\hat{N}}_{z}\right)}^{r}⊙{\hat{G}}_{x}\right)\right)+ & \\ \nabla_{y}^{*}\left({\left({\hat{N}}_{z}\right)}^{r}⊙\left({\left({\hat{N}}_{z}\right)}^{r}⊙\nabla_{y}Z_{min}-{\left({\hat{N}}_{z}\right)}^{r}⊙{\hat{G}}_{y}\right)\right)) & =0 \end{array}$$

The influence of varying *r* on the behavior of the surface orientation normal penalty function is shown in Figure 2. It can be seen that with r=0.5 the generalized method of Nehab penalizes steeper slopes stronger than the original method of Nehab (equivalent to r=1) and weaker than the gradient-based method (equivalent to r=0). On the other hand, with r=1.6 the proposed method exhibits a more tolerant behavior towards steeper inclination angles than the original method of Nehab, resulting in a global behavior that is reasonably close to the geodesic penalty in both the polar and lateral deviation directions. The value of the parameter *r* might still be further optimized.

Regarding the optimal choice of *r*, we have recognized that r=1.6 behaves in the vicinity to the geodesic distance. If the structure of the data shows strong noise on edges steeper than approximately 50 degrees, a lower choice of *r* can be considered.

### 4.7. Total Generalized Variation

In this section, we introduce another novel method to reconstruct a refined surface with a hybrid depth and surface normal approach that is based on a Total Generalized Variation (TGV) approach as introduced in [50]. The TGV is an extension of the Total Variation (TV), which is a very popular and efficient regularization technique used currently in many image processing applications, however, it is known for producing staircasing artifacts in slope regions of the solution. In contrast, the TGV overcomes the staircasing problem by allowing solutions of higher order. Our formulation is a gradient-based approach, which restricts the surface to be close with a quadratic penalty to an initial depth solution $\hat{Z}$, while enforcing the auxiliary gradient field G to be in the proximity of the given gradient field $\hat{G}$, propagated through the TGV penalty. Thereby, we simultaneously reconstruct the surface *Z* and the auxiliary gradient field G, such that our discrete model is given as follows:

(37) $$\min_{Z,G}\alpha_{1}||\nabla Z-{G||}_{2,1}+\alpha_{0}{\left||\nabla G|\right|}_{2,1}+\frac{\alpha }{2}||Z-\hat{Z}{\left|\right|}^{2}+\frac{\beta }{2}||G-\hat{G}{\left|\right|}^{2},$$

where the gradient operator $\nabla :R^{M\times N\times 2}\to R^{M\times N\times 4}$ computes finite differences and ∇G can be decomposed into $\left(\nabla G_{x},\nabla G_{y}\right)$, where ∇ was defined in Equation (4). Therefore, the first and second order components of our TGV regularization have the following form:

(38) $$||\nabla Z-{G||}_{2,1}=\sum_{i,j}\sqrt{{\left(\nabla_{x}Z\right)}_{i,j}^{2}+{\left(G_{i,j,x}\right)}^{2}+{\left(\nabla_{y}Z\right)}_{i,j}^{2}+{\left(G_{i,j,y}\right)}^{2}},\;\mathrm{and}$$

(39) $${\left||\nabla G|\right|}_{2,1}=\sum_{i,j}\sqrt{{\left(\nabla_{x}G_{x}\right)}_{i,j}^{2}+{\left(\nabla_{y}G_{x}\right)}_{i,j}^{2}+{\left(\nabla_{x}G_{y}\right)}_{i,j}^{2}+{\left(\nabla_{y}G_{y}\right)}_{i,j}^{2}}.$$

The function in Equation (37) consists of a depth and an orientation constraint as well as the TGV regularization terms. The depth constraint (i.e., the third term of Equation (37)) enforces the depth solution *Z* to stay in the vicinity of our noisy initial depth map $\hat{Z}$. The orientation constraint (i.e., the fourth term of Equation (37)) enforces G to stay in the proximity of the given surface gradient estimation $\hat{G}$. The TGV regularization part comprises a first and a second order term (first two terms of Equation (37)). The latter represents the TV component and penalizes the $l_{1}$-norm of the second order gradient field, which forces the gradient field G to be piecewise constant. The former enforces the approximated gradient of the depth map ∇Z to stay in the proximity of the auxiliary gradient field G also through an $l_{1}$ penalty.

Given our primal problem in Equation (37) we formulate a primal-dual (PD) problem, which belongs to the class of saddle-point problems, as follows:

(40) $$\min_{x}\max_{y}{\left(Kx\right)}^{T}y-F^{*}\left(y\right)+H\left(x\right),$$

where *H* describes the depth and orientation constraints, *F* defines the TGV component, with its convex conjugate $F^{*}$ and the linear operator *K* is defined as:

(41) $$K=\left(\begin{array}{cc} \nabla & -I\\ 0 & \nabla \end{array}\right)\;.\;$$

Our primal variable is denoted as $x\in R^{M\times N\times 3}$ and $y\in R^{M\times N\times 6}$ represents the dual variable:

(42) $$x=\left[\begin{array}{c} Z\\ G_{x}\\ G_{y} \end{array}\right]\;,\;\;y=\left[\begin{array}{c} y_{1}\\ y_{2} \end{array}\right]\;,\;\;y_{1}=\left[\begin{array}{c} y_{1,1}\\ y_{1,2} \end{array}\right]\;,\;\;y_{2}=\left[\begin{array}{c} y_{2,1}\\ y_{2,2}\;\\ y_{2,3}\\ y_{2,4} \end{array}\right].$$

where $y_{1}$ and $y_{2}$ hold the dual variables of the first and the second term in Equation (37) respectively, with $y_{1}$ holding two terms for each $\nabla_{x}$ and $\nabla_{y}$, while $y_{2}$ holds four terms, each for $\nabla_{x}$ and $\nabla_{y}$ in $G_{x}$ and $G_{y}$. For $F\left(x\right)={\alpha ||x||}_{p}$, where $\left|\right|\cdot {\left|\right|}_{p}$ is an $l_{p}$-norm, we calculate the convex conjugate as follows:

(43) $$F^{*}\left(y\right)=\delta_{\left|\right|\cdot {\left|\right|}_{2,\infty }\leq \alpha }\left(y\right)=\left\{\begin{array}{cc} 0, & \mathrm{if}\;|y_{i,j}{|}_{2}\leq \alpha ,\forall i,j\\ \infty , & \mathrm{otherwise}, \end{array}\right.$$

which is the indicator function of the polar ball. Therefore, $F^{*}$ and *H* are defined as follows:

(44) $$F^{*}\left(y\right)=\delta_{\left|\right|\cdot {\left|\right|}_{2,\infty }\leq \alpha_{1}}\left(y_{1}\right)+\delta_{\left|\right|\cdot {\left|\right|}_{2,\infty }\leq \alpha_{0}}\left(y_{2}\right),\;\mathrm{and}$$

(45) $$H\left(x\right)=\frac{\alpha }{2}||Z-\hat{Z}{\left|\right|}^{2}+\frac{\beta }{2}||G-\hat{G}{\left|\right|}^{2}.$$

An optimal solution to our hybrid formulation is found with the PD algorithm, as described in Appendix A.2. For a more detailed description of the PD algorithm, see [51].

## 5. Evaluation

To evaluate all considered algorithms we need two data structures, namely an initial depth map $\hat{Z}$ and surface orientation information such as surface normals $\hat{N}$ or gradients $\hat{G}$. Initial depth maps can be provided by stereo or light field correspondence analysis or other depth scanning methods. An estimate of the surface orientation is calculated using e.g., photometric stereo or polarization imaging. Our discussed hybrid algorithms differ mainly in how the surface orientation information is taken into account. In particular, the penalty functions associated with the surface orientation constraints vary significantly for different methods, as illustrated in Figure 1 through Figure 4c. We proposed two novel methods in Section 4.6 and Section 4.7, namely the generalized method of Nehab and the TGV approach. The former balances the surface orientation information with an improved weighting function withing the least squares framework. The latter uses a TGV regularization term combined with a gradient-based approach.

### Qualitative and Quantitative Evaluation

In order to perform a comprehensive quantitative evaluation of the above mentioned methods, we considered using synthetic evaluation data that comprises full ground truth (GT) information. The initial depth $\hat{Z}$ for the synthetic evaluation data is given by a ground truth depth map ${\hat{Z}}_{GT}$, which is thresholded after adding noise:

(46) $$\hat{Z}=\frac{1}{k}\left[k\cdot ({\hat{Z}}_{GT}+noise)\right].$$

In this study, we considered a normally distributed additive noise as this type of noise well simulates the behavior of matching errors of stereo or light field methods combined with image sensor noise. The noise used in our evaluations has a maximum amplitude of 7% of the depth range. The constant *k* defines the number of discretization steps and [·] rounds to the nearest integer number n∈Z. These discretization artifacts attempt to simulate the output of the discrete regularized correspondence analysis, which is often applied in real-world scenarios (e.g., as described in [37]).

For the orientation constraints, we assume surface normals $\hat{N}$ derived from the ground truth depth model ${\hat{N}}_{GT}$ by adding a normally distributed noise with a maximum amplitude of 23% of the normal range in the spherical coordinate system:

(47) $$\hat{N}=\frac{{\hat{N}}_{GT}+noise}{\left|\right|{\hat{N}}_{GT}+noise\left|\right|}$$

**Figure 5:**
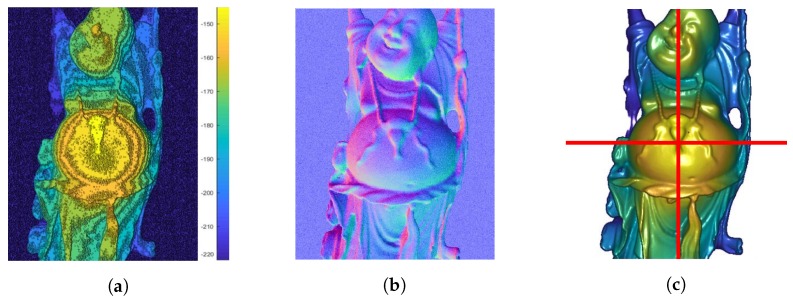
Example evaluation data, consisting of a noisy quantized depth $\hat{Z}$ and noisy surface normals $\hat{N}$. The ground truth depth ${\hat{Z}}_{GT}$ is shown with a horizontal and vertical cross-section line, which indicates the positions of evaluations in the following Figure 6. (**a**) Initial depth $\hat{Z}$; (**b**) Given surface normals $\hat{N}$; (**c**) Ground truth depth ${\hat{Z}}_{GT}$.

In Figure 5, examples of the evaluation data $\hat{Z}$ and $\hat{N}$ as well as the corresponding ground truth depth ${\hat{Z}}_{GT}$ are shown. All 3D datasets used for evaluations were taken from the Stanford 3D scanning repository [52] and rendered with POV-Ray [53].

**Figure 6:**
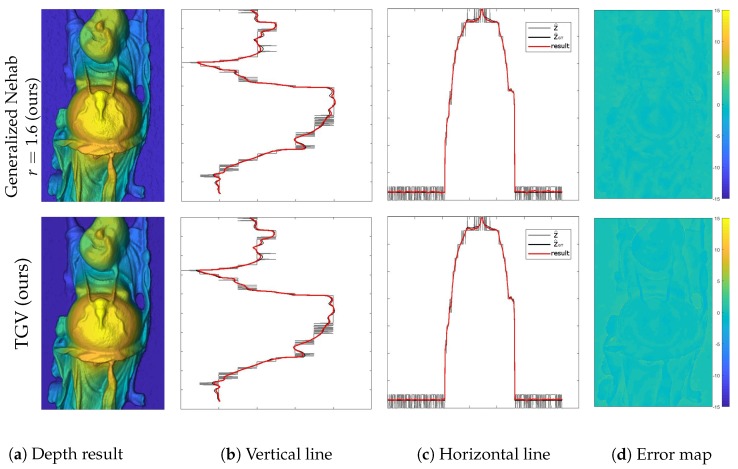
Qualitative evaluation of our novel approaches, as introduced in Section 4.6 and Section 4.7. The following methods are demonstrated: (1st row) the generalized method of Nehab with r=1.6, (2nd row) the TGV approach. The left column shows the color coded depth reconstructions as delivered by different methods. The middle two columns show the vertical and horizontal depth profiles as marked in Figure 5c. In each of these plots, the red, gray and black lines indicate the reconstructed depth *Z*, the initial depth $\hat{Z}$ and the ground truth depth ${\hat{Z}}_{GT}$, respectively. The error maps showing signed distances to the ground truth depth are provided in the right column.

**Figure 7:**
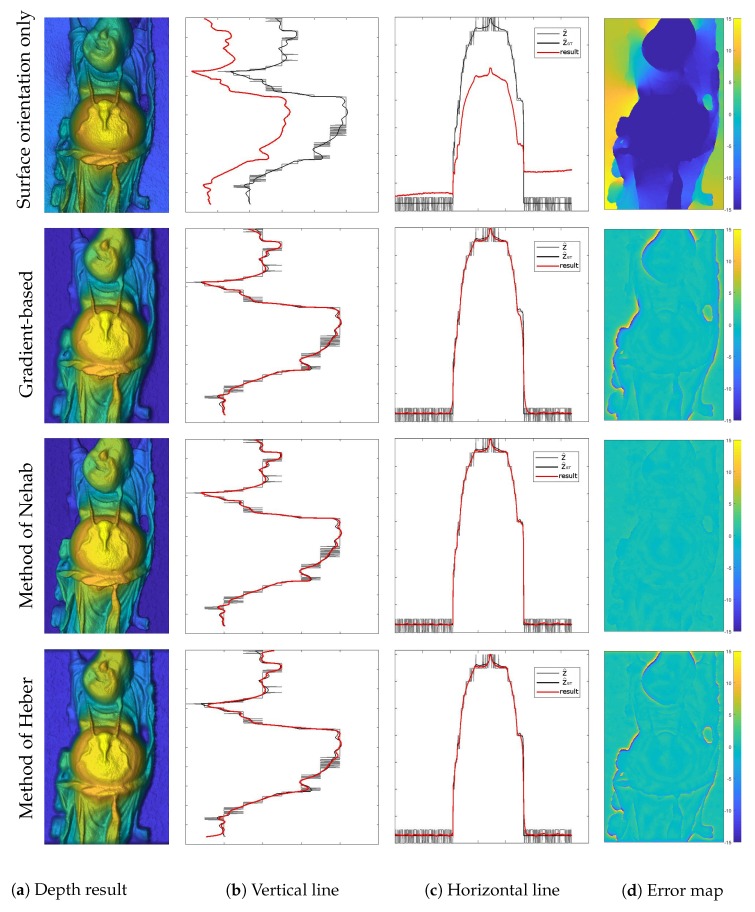
Qualitative evaluation of the state-of-the-art methods described in Section 4. The following methods are demonstrated: (1st row) the gradient-based method using surface orientations only, (2nd row) the combined gradient-based method, (3rd row) the method of Nehab, and (4th row) the method of Heber. The left column shows the color coded depth reconstructions as delivered by different methods. The middle two columns show the vertical and horizontal depth profiles as marked in Figure 5c. In each of these plots, the red, gray and black lines indicate the reconstructed depth *Z*, the initial depth $\hat{Z}$ and the ground truth depth ${\hat{Z}}_{GT}$, respectively. The error maps showing signed distances to the ground truth depth are provided in the right column.

**Figure 8:**
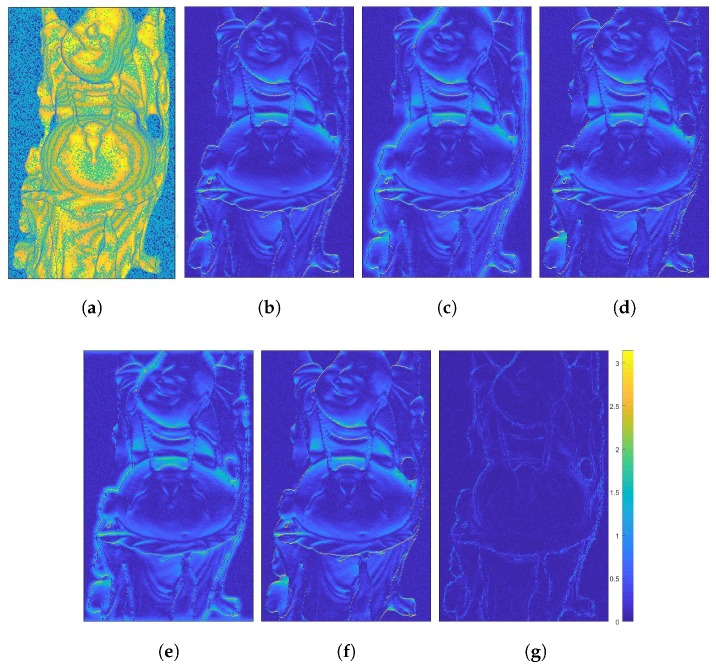
Geodesic distances of the ground truth normals to the normals from the initial depth map $\hat{Z}$ as well as the distances to the normal results of the presented methods (in the same order as displayed in Figure 6 and Figure 7). (**a**) Initial depth $\hat{Z}$; (**b**) Surface orientation only; (**c**) Gradient-based; (**d**) Method of Nehab; (**e**) Method of Heber; (**f**) Generalized Nehab r=1.6 (ours); (**g**) TGV (ours).

Qualitative comparisons of the methods described in Section 4 are presented in Figure 6 and Figure 7. The left column shows the color coded depth reconstructions as delivered by different methods. The middle two columns show the vertical and horizontal depth profiles as marked in Figure 5c. In each of these plots, the red, gray and black lines indicate the reconstructed depth *Z*, the initial depth $\hat{Z}$ and the ground truth depth ${\hat{Z}}_{GT}$, respectively. The error maps showing signed distances to the ground truth depth are provided in the right column. The corresponding geodesic distances from the ground truth surface normals ${\hat{N}}_{GT}$ for each method are displayed in Figure 8. Results of the quantitative evaluations are shown in Table 2 for gradient-based methods and in Table 3 for normal based methods. For the evaluation we used three datasets from [52]: Buddha (shown in qualitative analysis), Dragon, and Armadillo. The tables hold fractional precisions of two digits in the depth evaluation and a higher fractional precision of four digits in the normal evaluation due to different ranges. The average is calculated using 4 digits after the comma, rounding errors can cause differences in the last digit. The MSE distance in the depth domain is calculated by the quadratic distance between the ground truth depth and the depth result:

(48) $$MSE_{Z}=d_{Z}=\frac{1}{MN}\left|\right|{\hat{Z}}_{GT}-Z{\left|\right|}^{2}$$

The geodesic distance is calculated as described in Section 4.1 by using the following equation:

(49) $$GEO_{N}=d_{N}=\frac{1}{MN}\sum_{i,j}^{MN}acos\left(\frac{\langle {\hat{N}}_{i,j},\left(-{\left(\nabla_{x}Z_{GT}\right)}_{i,j},-{\left(\nabla_{y}Z_{GT}\right)}_{i,j},1\right)\rangle }{|\left(-{\left(\nabla_{x}Z_{GT}\right)}_{i,j},-{\left(\nabla_{y}Z_{GT}\right)}_{i,j},1\right){|}_{2}}\right)$$

As can be seen in Figure 7(1st row) and Figure 8b, using surface orientation information alone in the gradient-based formulation provides visually pleasing detail reconstructions ($d_{N}=0.2365$), though it is performing worst in terms of the absolute distance to the ground truth depth with an average MSE of $d_{Z}=66.68$. In Figure 7(2nd row) and Figure 8c, one can see that adding a depth constraint to the same gradient-based formulation improves the result drastically ($d_{Z}=2.04$ and $d_{N}=0.2951$). Nevertheless, this method still shows somewhat low performance around steep edges. Note that each of the demonstrated methods could be used to reconstruct surfaces from surface normals only solely by dropping the depth term. The method of Nehab shown in Figure 7(3rd row) and Figure 8d exhibits the capability to improve the result over the previous methods exploiting a better surface orientation weighting strategy ($d_{Z}=0.14$, $d_{N}=0.2532$). These methods are optimized using a least squares solver. The evaluation of Heber’s method is shown in Figure 7(4th row) and Figure 8e. The method is optimized using gradient descent and reaches average results of $d_{Z}=1.79$ and $d_{N}=0.3049$, which is significantly worse than the method of Nehab. The results of our generalized method of Nehab with a parametrization r=1.6 are shown in in Figure 6(1st row) and Figure 8f. This method improves robustness over the standard method of Nehab against noise in surface normals and outperforms all other evaluated methods in the absolute depth error domain with $d_{Z}=0.11$. As the normal weighting here is in closer vicinity to the geodesic penalty than the method of Nehab, this approach reaches an improved normal error of $d_{N}=0.2442$. This method is optimized using a least squares solver. Our novel TGV method, shown in Figure 6(2nd row) and Figure 8g, provides by far the best normal accuracy of $d_{N}=0.0666$ and performs among the best in the depth domain ($d_{Z}=0.20$). It is optimized with a primal-dual algorithm.

For completion, we additionally show the results of two regularized gradient methods in Table 3. First, we regularize with smoothness as shown in Equation (23), which can be used for sparse data. In our dense case, where we used scalars for the weighting parameters, the normal accuracy shows a minor improvement due to smoothing in return of a weaker depth accuracy. Second, we regularize with the gradient, as shown in Equation (21). With this we can reach an improvement both in the depth and normal accuracy. These regularization terms could be used for all presented methods, which would exceed the scope of this paper. Note that our novel TGV method shows a significantly better performance in both accuracy measurements.

**Figure 9:**
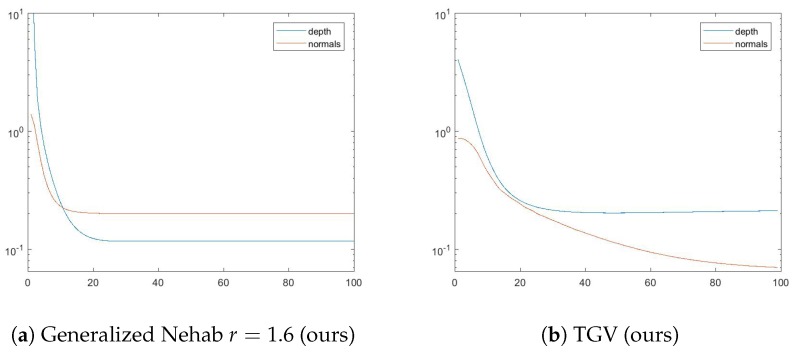
Convergence analysis of the two winning methods: the generalized Nehab with r=1.6 (**a**) and the TGV (**b**). Distances to the ground truth depth and surface normals are shown after each iteration, plotted with a logarithmic *y*-axis for better visibility.

As can be seen in Table 2 and Table 3, the two best performing methods are our generalized method of Nehab with r=1.6 (normal based) and our novel TGV method (gradient-based). We show the convergence of both in Figure 9 with respect to the depth error defined in Equation (48) and the normal error as defined in Equation (49). While the generalized Nehab converges in both terms after approximately 25 iterations, the TGV settles at around the same iteration step with a depth error which is performing slightly worse than the other method, but continues to highly optimize the normal error. Note that a gradient-based formulation was chosen to demonstrate graceful properties of our novel TGV approach. It significantly improved the results compared with the standard gradient-based method. Alternatively, for even better performance, other penalty functions such as the generalized method of Nehab, can be chosen and will be a matter of future research.

**Figure 10:**
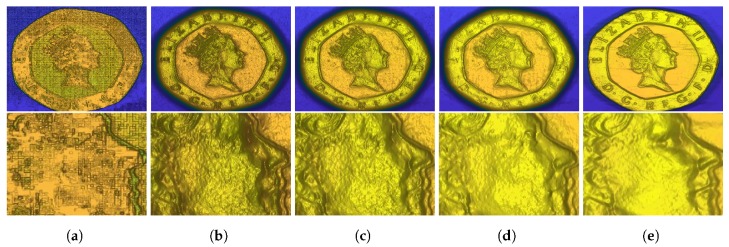
Real world evaluation of a coin object acquired by a multi-line scanner. The acquisition setup was previously described in detail in [37]. (**a**) $\hat{Z}$; (**b**) Gradient-based; (**c**) Method of Nehab; (**d**) Generalized Nehab (ours); (**e**) TGV (ours).

We demonstrated the described algorithms with different surface orientation weighting on a real world example, as shown in Figure 10. This object was acquired with a multi-line scanner, the hybrid light field - photometric stereo acquisition framework described in [37]. In this very specific case we only have one normal direction. An example how to deal with this data in the gradient-based approach is weighting the gradient vector in Equation (23) in the missing direction with zero. Specifics of the multi-line-scanning environment are out of the scope of this paper and are a matter of future research.

## 6. Conclusions

We presented a review and classification of methods combining depth and surface orientation data (normals or gradients), in order to reach an improved surface depth estimation. State-of-the-art methods differ mostly in the formulation of the surface orientation constraint (see Section 4) and capabilities of the method-specific solvers.

We illustrated the differences between various formulations of the surface orientation constraint and explained performance discrepancies. Furthermore, we used our findings to introduce a generalization of the method of Nehab (see Section 4.6) that significantly outperforms other methods in terms of absolute depth accuracy. Additionally, we introduced a novel method based on TGV (see Section 4.7), which outperforms all other methods in the surface normal domain and shows a competitive performance in the depth accuracy. While our generalized Nehab method converges faster (see Figure 9) and gives the most accurate result in the depth domain, our TGV based approach refines the surface orientation further and converges at the most accurate orientation result with a high accuracy in the depth domain.

Further research will include the specialization on line-scanning algorithms, TGV weighting adaption and computational acceleration. With specialized hybrid algorithms that fit data from line scanning sensors we will determine a solution with incomplete surface orientation data. The surface orientation constraint of our TGV formulation is currently gradient-based. Plugging in another formulation with a better balanced normal weighting could improve the results even further and will be a matter of future research. Furthermore we will focus on computational acceleration of the proposed algorithms, where we will exploit their inherent structure to achieve efficient parallelization.

## Figures and Tables

**Table 1:** Overview of the presented methods. The behavior of the surface orientation constraint in regions with different orientations is visualized for each method in Figure 1 and Figure 2.

Method	Depth Penalty	Orientation Penalty	Balance at Flat Regions	Balance at Steep Regions
Surface orientation only	✗	Gradient-based	✓	✗
Gradient-based	✓	Gradient-based	✓	✗
Gradient-based	✓	Gradient-based + regularization with zero Laplace (Equation (23))	✓	✗
Gradient-based	✓	Gradient-based + regularization with gradient (Equation (21))	✓	✗
Method of Heber	✓	Scaled normal	✓	✗
Method of Nehab	✓	Projection of surface tangents to the given normal field	✓	∽
Generalized Nehab (ours)	✓	Projection of surface tangents to the given normal field with additional $N_{z}$ weighting	✓	✓
TGV (ours)	✓	Gradient-based + TGV	✓	✓

**Table 2:** Gradient-based: quantitative evaluation of the distance of the optimized depth values to the ground truth depth. The evaluations were performed on objects from the Stanford database [52], which were rendered using POV-Ray [53]. Evaluated are the MSE to the ground truth depth and the geodesic distance to the ground truth normals.

Gradient-Based	Dataset Method	$\hat{Z}$	Surface Orientation Only	Gradient Based	Gradient Based + Reg. with Laplacian Smoothness (Equation (23))	Gradient Based + Reg. with Gradient (Equation (21))	TGV (Ours)
Depth [$MSE_{Z}$]	Dragon	4.23	34.05	2.04	2.15	1.85	**0.19**
	Buddha	4.85	117.29	2.12	2.25	2.01	**0.22**
	Armadillo	4.60	48.71	1.95	2.06	1.83	**0.18**
	Average	4.53	66.68	2.04	2.15	1.90	**0.20**
Normals [$GEO_{N}$]	Dragon	0.8226	0.2776	0.3344	0.3200	0.3017	**0.0664**
	Buddha	0.8767	0.1922	0.2535	0.2339	0.2125	**0.0668**
	Armadillo	0.8611	0.2397	0.2973	0.2797	0.2599	**0.0666**
	Average	0.8535	0.2365	0.2951	0.2779	0.2580	**0.0666**

**Table 3:** Normal based: quantitative evaluation of the distance of the optimized depth values to the ground truth depth. The evaluations were performed on objects from the Stanford database [52], which were rendered using POV-Ray [53]. Evaluated are the MSE to the ground truth depth and the geodesic distance to the ground truth normals.

Normal Based	Dataset Method	$\hat{Z}$	Method of Heber	Method of Nehab	Generalized Nehab r=1.6 (Ours)
Depth [$MSE_{Z}$]	Dragon	4.23	2.01	0.13	**0.10**
	Buddha	4.85	1.60	0.15	**0.12**
	Armadillo	4.60	1.75	0.13	**0.10**
	Average	4.53	1.79	0.14	**0.11**
Normals [$GEO_{N}$]	Dragon	0.8226	0.3474	0.2941	**0.2849**
	Buddha	0.8767	0.2579	0.2102	**0.2013**
	Armadillo	0.8611	0.3094	0.2553	**0.2464**
	Average	0.8535	0.3049	0.2532	**0.2442**

## Abbreviations

The following abbreviations are used in this manuscript:

CRF	Conditional Random Field
GT	Ground Truth
FISTA	Fast Iterative Shrinkage Thresholding Algorithm
ISTA	Iterative Shrinkage Thresholding Algorithm
MRF	Markov Random Field
MSE	Mean Squared Error
PD	Primal-Dual
SfF	Shape from Focus
SfM	Structure from Motion
TGV	Total Generalized Variation
ToF	Time of Flight
TV	Total Variation

## Appendix A. Optimization Theory

In this section we show two advanced optimization methods used to fuse depth and surface orientation as well as the type of functions they can optimize.

### Appendix A.1. Accelerated Proximal Gradient Method

The fast iterative shrinkage thresholding algorithm (FISTA) minimizes an objective function by using proximal operators (see [54]). It works on general conditions as non-smooth convex functions and can be very fast, as there exist simple proximal operators for various energy functions. Proximal gradient methods consider optimization problems with the objective split in two components:

(A1) $$\min_{x}F\left(x\right)+H\left(x\right),$$

where $F:R^{N}\to R$ and $H:R^{N}\to R\cup \left\{+\infty \right\}$ are closed proper convex functions and where one of the functions (*F*) is additionally differentiable. The proximal gradient problem uses a step size $\lambda^{k}>0$ and is denoted as follows:

(A2) $$x^{k+1}\leftarrow prox_{\lambda^{k}H}\left(x^{k}-\lambda^{k}\nabla F\left(x^{k}\right)\right).$$

If ∇F is Lipschitz continuous with a constant *L* and a fixed step size $\lambda^{k}=\lambda \in \left(0,1/L\right]$ is used, the method converges in O(1/k). Accelerated proximal gradient methods have an additional extrapolation step in the algorithm and are denoted as follows:

(A3) $$\begin{array}{ccc} y^{k+1} & \leftarrow & x^{k}+w^{k}\left(x^{k}-x^{k-1}\right), \end{array}$$

(A4) $$\begin{array}{ccc} x^{k+1} & \leftarrow & prox_{\lambda^{k}H}\left(y^{k+1}-\lambda^{k}\nabla F\left(y^{k+1}\right)\right), \end{array}$$

where $\lambda^{k}$ denotes the step size and $w^{k}$ an extrapolation parameter which is described as:

(A5) $$w^{k}=\frac{k-1}{k+2}.$$

FISTA is one example of such an accelerated proximal gradient method and described in detail in [49]. In [41], the FISTA algorithm is used to optimize the method of Heber, which we discuss in Section 4.4.

### Appendix A.2. Primal-Dual

Primal-dual methods can be used to solve a large number of optimization problems. The considered saddle-point problem, which is a primal-dual formulation has the following primal form:

(A6) $$\min_{x\in X}F\left(Kx\right)+H\left(x\right).$$

The corresponding saddle point problem is described as:

(A7) $$\min_{x\in X}\max_{y\in Y}\langle Kx,y\rangle +H\left(x\right)-F^{*}\left(y\right).$$

We are using the Chambolle-Pock [55] primal-dual method and compute the proximal maps for *H* and $F^{*}$. A proximal descent is computed in the primal variable *x* and, in an alternating manner, a proximal ascent in the dual variable *y*:

(A8) $$\begin{array}{c} \left\{\begin{array}{c} x^{k+1}\leftarrow {\mathrm{prox}}_{\tau H}\left(x^{k}-\tau K^{T}y^{k}\right)\\ y^{k+1}\leftarrow {\mathrm{prox}}_{\sigma F^{*}}\left(y^{k}+\sigma K\left(2x^{k+1}-x^{k}\right)\right) \end{array}\right. \end{array}$$

The convergence rate is O(1/N), and can be improved if *H* or $F^{*}$ are uniformly convex to $O(1/N^{2})$. Both, primal and the dual being uniformly convex the algorithm shows a linear convergence $O(e^{N})$ [55,56,57]. We discuss the primal-dual optimization on our hybrid TGV formulation in Section 4.7.
